# The RE(ACT) Initiative and the use of an online community to enhance research on rare diseases

**DOI:** 10.1186/1750-1172-9-S1-P8

**Published:** 2014-11-11

**Authors:** Olivier Menzel

**Affiliations:** 1 BLACKSWAN Foundation, Chemin de la Riaz 11, 1418 Vuarrens, Switzerland

## 

There is a striking need for increased international cooperation in scientific research on rare diseases (RDs). Existing research efforts are in fact still scattered and fragmented research is being performed with little coordination between research laboratories. This lack of coordination is particularly detrimental to the increase of knowledge and to the delivery of new therapies for RDs because the resources are very limited and the patient population is small for each disease. Moreover, traditional funding mechanisms based on natural market conditions and access to public funds, do not fit the reality of research requirements on RDs.

For these reasons the BLACKSWAN Foundation is proud to present the RE(ACT) Initiative with the aim of boosting research and facilitating the discovery of new molecules and therapies for millions of patients. The Initiative is structured on two main axes: the RE(ACT) Congress (react-congress.org) and the online RE(ACT) Community (react-community.org). Their mission is to strengthen the synergies between researchers and other stakeholders that are related to different extents with RDs.

The RE(ACT) Congress is organized every two years and brings together world leaders and young scientists from different scientific fields to present state-of-the-art research, to discuss results and to exchange ideas.

The RE(ACT) Community as an example of how the use of online communities and social media can address these challenges and facilitate the cooperation between researchers, patients and other important stakeholders in the RDs field. The RE(ACT) Community uses an innovative online platform that combines elements of scientific knowledge sharing with the access to new funding mechanisms to improve the implementation of research projects. This approach is conceived to empower researchers and patients and change the way science is communicated. The RE(ACT) Community is a place where everybody has an equal opportunity to seek, share and generate knowledge. Patients and patient organizations have the possibility to exchange their observations on a disease directly with researchers and researchers can be put in contact with patients who express the interest to take part in clinical trials, provide samples, or support. Moreover, crowdfunding is introduced as original financing method to support research in the field of RDs as it involves funding a project with relatively modest contributions from a large group of individuals. The promotion of a research project can be facilitated by social media through the integration of “social plugin” on platforms, which allow a user to sensitize his network and improve awareness on a specific research or disease.

The RE(ACT) Community is a new kind of collaborative workspace where RD stakeholders help each other to develop research and increase awareness in the public opinion on the subject of RDs.

